# Prefrontal activation and connectivity during verbal fluency in autism Spectrum disorder: an fNIRS study

**DOI:** 10.3389/fnhum.2026.1810519

**Published:** 2026-07-24

**Authors:** Bin Yu, Xi-Ning He, Xiao-Hong Zhang, Ge Lei, Xuan-Yue Song, Jing-Li Wu, Ding-Xu Li, Shu-Ning Cheng, Yi Zhang, Jian-Min Lv

**Affiliations:** 1 Pediatric Medical Rehabilitation Center, Shaanxi Provincial Rehabilitation Hospital, Xi'an, Shaanxi, China; 2 Department of Child Health, Hanzhong Central Hospital, Hanzhong, Shaanxi, China; 3 Department of Child Health, The People's Hospital of Lantian County, Xi'an, Shaanxi, China; 4 Rehabilitation Science Institute, Shaanxi Provincial Rehabilitation Hospital, Xi'an, Shaanxi, China

**Keywords:** autism spectrum disorder (ASD), cortical activation, executive function (EF), functional connectivity, functional near-infrared spectroscopy (fNIRS), verbal fluency task (VFT)

## Abstract

**Objective:**

The neural mechanisms behind varying cognitive ability in children with Autism Spectrum Disorder (ASD) remain unclear. This study compared cortical activation and brain connectivity across ASD subgroups stratified by Full-Scale Intelligence Quotient (FSIQ).

**Methods:**

Sixty-four children with ASD were divided into two groups based on their FSIQ scores: Group 1 (*n* = 30, FSIQ ≥ 70) and Group 2 (*n* = 34, FSIQ < 70). Using a 19-channel functional near-infrared spectroscopy (fNIRS) system, we assessed cortical activation and functional connectivity in both groups during a Verbal Fluency Task (VFT) by monitoring changes in oxyhemoglobin (Oxy-Hb) concentration. Inter-group differences in brain activation and connectivity were compared, and correlations between cortical activation levels and clinical indices (FSIQ, verbal production) were examined.

**Results:**

Compared to Group 2, Group 1 showed significantly stronger cortical activation in channels 1 and 2 [corresponding to the inferior frontal gyrus (IFG)] (*U* = 212.000*, Z* = −4.009, *FDR-corrected p* < 0.001*; U* = 160.000, *Z* = −4.709, FDR-corrected *p* < 0.001). Global functional connectivity was also greater in Group 1 (mean = 0.586, *SD* = 0.521) relative to Group 2 (mean = 0.413, *SD* = 0.521) (permutation statistic = 4.82, FDR-corrected *p* < 0.001). Moreover, Oxy-Hb changes in channels 1 (*r_s_* = 0.304, *p* < 0.05), 2 (*r_s_* = 0.405, *p* < 0.01), and 10 (*r_s_* = 0.253, *p* < 0.05) were positively correlated with FSIQ scores. Activation in channels 1 (*r_s_* = 0.529, *p* < 0.001), 2 (*r_s_* = 0.569, *p* < 0.001) and 10 (*r_s_* = 0.290, *p* < 0.05) were also significantly correlated with verbal production.

**Conclusion:**

Our findings provide neurofunctional evidence for the association between cognitive resource capacity and verbal-executive processing in ASD. ASD children with lower FSIQ exhibited lower prefrontal activation and lower functional connectivity during the VFT, particularly within the IFG, frontopolar cortex (FPC), and dorsolateral prefrontal cortex (DLPFC). These results underscore the utility of fNIRS in delineating distinct neurocognitive profiles within ASD and highlight the IFG as a key region where neural activity correlates with both cognitive ability and language output, offering a potential target for mechanism-based intervention strategies.

## Introduction

Autism spectrum disorder (ASD) is a complex and heterogeneous neurodevelopmental condition characterized by challenges with communication, social interaction, and behavior (Audras-Torrent et al., 2021; Genovese and Butler, 2020). According to the World Health Organization, approximately 1 in 100 children are diagnosed with ASD (Elsabbagh et al., 2022). Approximately 30–50% of individuals with ASD exhibit co-occurring intellectual disability, which contributes to marked heterogeneity in cognitive and language profiles (He et al., 2025). The rising prevalence of ASD in recent years exerts a substantial impact on families and society, profoundly affecting the mental health and developmental trajectories of diagnosed individuals (Salari et al., 2022; Wang et al., 2023).

A core challenge in understanding ASD lies in its pronounced heterogeneity, particularly in the dissociation between intellectual ability and social-communicative function. This variability is closely tied to challenges with executive function and verbal fluency — key capacities for social adaptation. Verbal fluency itself integrates processes like vocabulary access and reasoning, which are essential for effective communication (Abdi et al., 2023). Neuroimaging studies further indicate altered brain function in ASD, including lower prefrontal-parietal connectivity and atypical prefrontal activation during cognitive tasks (Cheng et al., 2024; Parellada et al., 2023). However, how these neural mechanisms differentially support verbal fluency in children with varying levels of overall cognitive ability (often indexed by Full-Scale Intelligence Quotient, FSIQ) remains poorly understood. This gap hinders the development of tailored interventions (Andreou et al., 2025; Su et al., 2025).

Functional near-infrared spectroscopy (fNIRS) is a non-invasive neuroimaging method that measures brain activity by detecting changes in hemoglobin concentration (Zhang and Roeyers, 2019). It offers higher temporal resolution than fMRI and better tolerance to motion artifacts compared with other neuroimaging techniques. fNIRS is increasingly used to study brain function in psychiatric disorders, including in tasks involving executive function (Hosokawa et al., 2015; Pinti et al., 2020; Yeung and Lin, 2021). The Verbal Fluency Test (VFT) is a paradigmatic task that probes the interface between language and executive function. It requires not only the retrieval of words from semantic memory (a language-related process) but also the continuous engagement of cognitive control, including mental set-shifting between subcategories, inhibition of previously uttered or irrelevant words, and online monitoring of output against the given rule. Thus, VFT performance reflects the efficiency of verbal-executive integration. Functional near-infrared spectroscopy (fNIRS) is particularly suitable for studying such dynamic processes in children. Crucially, successful performance on complex tasks like VFT is supported by broad cognitive resources, which are commonly indexed by the Full-Scale Intelligence Quotient (FSIQ). FSIQ reflects integrated cognitive resources including attention control, working memory, reasoning, and processing efficiency, which collectively provide the foundational capacity for performing complex cognitive-linguistic tasks such as the VFT (Chen et al., 2025; Tan et al., 2025). The VFT is described here as a verbal-executive task because it simultaneously draws on both verbal abilities (lexical retrieval and word production) and executive functions (cognitive control, set shifting, inhibition, and online performance monitoring).

Therefore, comparing ASD children stratified by FSIQ during a VFT allows us to investigate how differences in overall cognitive resource availability are manifested in the neural underpinnings of verbal-executive processing (Husain et al., 2021; Whiteside et al., 2016).

Considering that both fNIRS and VFT are well-established tools in cognitive neuroscience, their integration provides an opportunity to study brain activation and connectivity during verbal fluency tasks. This approach can yield valuable insights into the neural mechanisms underlying verbal fluency and executive function in children with ASD (Hosokawa et al., 2015; Yeung and Lin, 2021). Previous studies based on fNIRS have shown that, during the VFT, individuals with ASD and co-occurring depression exhibit lower activation in widespread anterior temporal lobe regions compared to typically developing adolescents (Ohtani et al., 2021). However, the combined use of fNIRS and VFT to systematically investigate neural differences in relation to cognitive ability strata within ASD populations has been limited. This approach has not been fully explored, particularly in understanding how neural activation, functional connectivity, and their associations with behavioral output differ between children with varying levels of cognitive and verbal ability (Conti et al., 2022).

In this study, we aim to address this gap by using multi-channel fNIRS to investigate differences in prefrontal brain activation and network connectivity between children with ASD who have a Full-Scale Intelligence Quotient (FSIQ) ≥ 70 and those with a FSIQ < 70 during a VFT. Crucially, we will not only compare group-level neural responses but also examine how these neural measures correlate with both cognitive capacity (FSIQ) and overt behavioral performance (verbal output). This combined approach will help us to better understand the neural correlates of Cognitive ability and executive function in ASD, which could inform more targeted interventions (Al Ghamdi and AlMusailhi, 2024).

## Materials and methods

### Subjects

The minimum sample size required was fifty-two basedon G*Power (effect size: 0.80, alpha error: 0.05, and 80% power). Sixty-four children (6–12 years old) were recruited. Children aged 6–12 years were selected because this period represents a critical stage for prefrontal cortex maturation, language development, and executive function refinement. fNIRS is highly feasible and tolerable in this age group (Maalouf et al., 2024). The inclusion criteria were as follows: participants with a clinical diagnosis of Autism Spectrum Disorder (ASD) confirmed according to the DSM-5 criteria. To ensure a clinically significant level of symptom severity, a total score ≥ 30 on the Childhood Autism Rating Scale (CARS, 15-item version) was required. All participants had stable clinical conditions confirmed by medical records. The exclusion criteria were as follows: (1) a comorbid diagnosis of Attention-Deficit/Hyperactivity Disorder (ADHD); (2) known genetic syndromes (e.g., Fragile X syndrome, Rett syndrome, Tuberous Sclerosis Complex) or congenital metabolic disorders; (3) significant sensory difference (e.g., blindness, deafness); (4) a history of severe neurological insult (e.g., traumatic brain injury, epilepsy with generalized tonic–clonic seizures); or (5) any other major medical or psychiatric condition that could independently account for the observed level of intellectual disability or significantly confound the interpretation of brain-behavior relationships. These criteria were implemented to obtain a sample where the primary driver of intellectual and functional variability was more likely to be idiopathic ASD. No participants were nonverbal or minimally verbal; all used phrase speech. All participants underwent an intellectual assessment and core symptom evaluation for ASD, using the Wechsler Intelligence Scale for Children-Fourth Edition (WISC-IV). The WISC-IV yields four primary index scores: Verbal Comprehension (VCI), Perceptual Reasoning (PRI), Working Memory (WMI), and Processing Speed (PSI). The Full-Scale Intelligence Quotient (FSIQ) is derived from these four composite indices (Cha et al., 2025; Matsudaira et al., 2025). Verbal IQ mainly reflects language comprehension and verbal reasoning ability, while nonverbal IQ represents visual perception, spatial processing and logical reasoning capacity. Participants were stratified by their Full-Scale Intelligence Quotient (FSIQ) into two groups, using a cutoff of 70. This threshold aligns with the DSM-5 and ICD-11 diagnostic cutoff for Intellectual Disability (Intellectual Developmental Disorder) and is widely used in ASD research to separate subgroups with and without concurrent cognitive impairments (Howard et al., 2025; Kehrer-Sawatzki et al., 2020; Koko et al., 2025). The present study used FSIQ = 70 as a cutoff for stratifying cognitive levels and did not evaluate adaptive functioning. Group 1 comprised children with FSIQ ≥ 70 (*n* = 30), and Group 2 comprised those with FSIQ < 70 (*n* = 34). The assessments were conducted by trained doctoral-level interviewers to evaluate intellectual levels and autism symptoms.

A total of 64 participants with autism were recruited between April 2024 and March 2025 from the inpatient units of Shaanxi Provincial Rehabilitation Hospital, with 30 in Group 1 and 34 in Group 2. These children received inpatient admission for routine developmental assessment and rehabilitation treatment. All applicants underwent pre-recruitment screenings to evaluate their adaptability to the fNIRS equipment and task completion ability. Our core data quality control rule was implemented after data collection: participants with severe noise and motion artifacts across multiple recording channels would be completely excluded from subsequent analysis.

In the current study, all 64 enrolled children with autism successfully completed the whole verbal fluency task. After comprehensive signal inspection, none of the participants met the full-subject exclusion standard, so all 64 participants were retained for formal statistical analysis, and no participant was excluded entirely due to unsatisfactory data quality (approval number: 2025126). Poor-signal candidates were filtered out at pre-recruitment screening stage, and no participant exhibited uncorrectable widespread multi-channel artifacts after data collection. The remaining datasets showed minimal residual motion noise, and no further additional correction was applied to avoid potential overcorrection bias. All valid participants retained complete and valid 19-channel fNIRS signals for subsequent statistical analysis.

### Verbal fluency task

**Figure 1:**
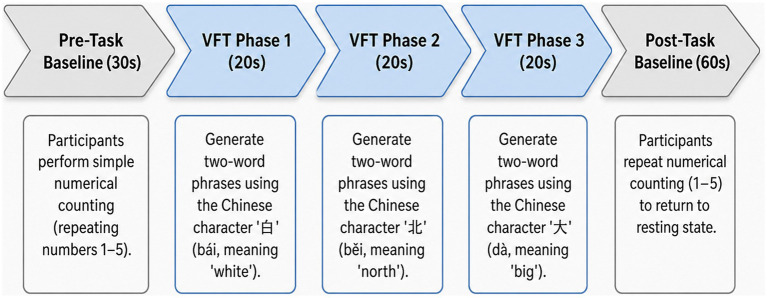
Schematic of the verbal fluency task (VFT). The task sequence included a 30-s pre-task baseline (numerical counting), three 20-s VFT phases (generating two-word phrases with the characters “白,” “北,” and “大”), and a 60-s post-task baseline (numerical counting).

A schematic timeline of the VFT is shown in Figure 1. The VFT was conducted during the daytime. It included a 30-s pre-task baseline block, a 60- s task period, and a 60-s post-task baseline block. For signal calibration, we extracted the 0–10 s stable window within the pre-task baseline to calculate the baseline hemodynamic level. Participants counted silently from 1 to 5 repeatedly during baseline periods (Liang et al., 2025). The 60 s post-task baseline was used to ensure complete hemodynamic signal recovery to baseline levels, which is a standard procedure in block-design fNIRS studies. During the task period, participants were asked to create as many phrases as possible using three common characters, such as “白” (white), “北” (north), and “大” (big). These three characters changed every 20 s to minimize the duration of silence. Participants were instructed to generate corresponding two-character Chinese phrases based on each cue character. We established clear criteria to classify responses during the verbal fluency task (VFT). Correct responses were defined as standard two-character Chinese phrases closely related to the given cue character. Taking the cue 白 (bái, white) as an example, typical valid answers included 白云 (bái yún, white cloud) and 白色 (bái sè, white colour). Responses were categorized as incorrect or excluded if they were single characters, repeated words, meaningless utterances, or phrases irrelevant to the cue. For instance, merely stating the single character 白 (bái, white), repeating the same phrase multiple times, or producing unrelated words such as 山水 (shān shuǐ, mountains and rivers) were all judged as invalid responses and excluded from subsequent behavioral analysis.

To ensure consistent linguistic and cognitive load across trials, all Chinese characters used were high-frequency words selected from the *Modern Chinese Common Character List*. The characters were monosyllabic, structurally simple, contained no polyphonic or rare forms, and could either stand alone as words or readily combine into common two-character words. Within each task block, the three characters were balanced for semantic category, part of speech, and phonological structure to minimize systematic bias from specific linguistic features. The character set was changed every 20 s to sustain task novelty, maintain cognitive load, and reduce attentional decline or lexical exhaustion due to prolonged exposure to the same stimuli.

### fNIRS measurement

Participants were comfortably seated in a quiet room, and hemoglobin concentrations were measured using a multi-channel near-infrared optical imaging system (NirSmart-6000A equipment, Danyang Huichuang Medical Equipment Co., Ltd., China). The sampling frequency was 11 Hz, and the wavelengths used were 730 nm and 850 nm. The probe array was positioned according to the international 10–20 system, with the middle probe centered on the FPz location. This configuration resulted in 19 measurement channels covering the prefrontal cortex. Most inpatients underwent the fNIRS evaluation within three days of hospitalization.

**Figure 2:**
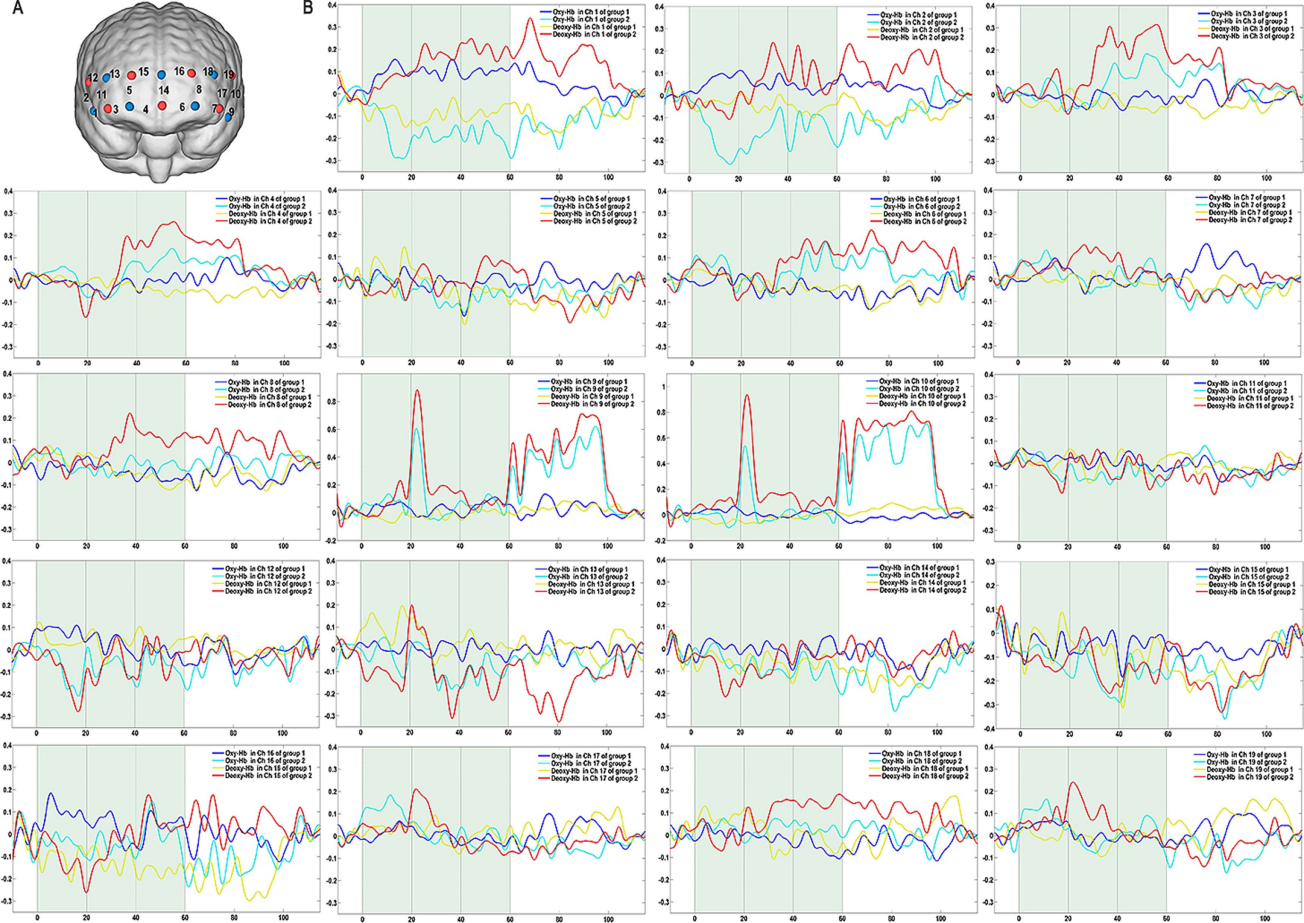
fNIRS channel montage and task-related hemodynamic responses across all 19 channels during the VFT. **(A)** Dorsal view of the 19 measurement channels registered to the standard MNI brain atlas. Red dots indicate emitters, and blue dots indicate detectors. Channels are mapped to different prefrontal subregions: IFG (Ch1, 2, 9); frontopolar area (Ch3–8, 11, 13–18); pars triangularis (PT) of Broca’s area (Ch10); and DLPFC (Ch12, 19). **(B)** All grand-averaged time courses of Oxy-Hb and Deoxy-Hb concentration changes across the 19 channels for Group 1 (FSIQ ≥ 70) and Group 2 (FSIQ < 70) are displayed in a 4 × 5 grid layout, with one cell left blank for orderly arrangement. The shaded area in each subplot represents the task period. Ch, Channel. Each waveform subplot uses identical axes: X-axis = Time from task onset (seconds); Y-axis = Hemoglobin concentration change (μM). All quantitative panels follow standardized axis labeling as described above.

The spatial registration of fNIRS channels to corresponding cerebral regions was performed using a standard 10–10 system template. The estimated Montreal Neurological Institute (MNI) coordinates for each channel were derived based on the known source-detector probe geometry and their placement relative to the cranial landmarks (nasion, inion, left and right pre-auricular points). These coordinates were then referenced to a validated brain atlas to associate each channel with its most probable underlying Brodmann area and anatomical gyrus, as summarized in the channel description above (see also Figure 2A). This template-based co-registration approach is widely adopted in fNIRS group studies, providing a standardized framework for localization. The spatial registration of all 19 fNIRS measurement channels was performed using the AtlasViewer software, with the adult Montreal Neurological Institute (MNI) template and the Automatic Anatomical Labeling (AAL) atlas. For each channel, the primary anatomical location was defined as the brain region with the highest coverage percentage. Detailed anatomical information for each channel is provided in Supplementary Table S1.

### Data processing and analysis

Prior to correlation analysis, Shapiro–Wilk normality tests were conducted for all continuous variables. Most variables were non-normally distributed, and only Oxy-Hb in Ch1, Ch2, and Ch17 were normally distributed (all *p* > 0.05). Therefore, Spearman rank correlation was used for all correlation analyses. Detailed results are presented in Supplementary Table S8.

We utilized the NirSpark software package (HuiChuang, China) to analyze NIRS data. First, to anatomically localize the fNIRS channels, spatial registration was performed. The three-dimensional coordinates of each source and detector probe were estimated based on their known distances from cranial landmarks (nasion, inion, left and right pre-auricular points). These coordinates were then registered to the standard Montreal Neurological Institute (MNI) space using a template head model. Each channel was subsequently mapped to its most probable corresponding Brodmann area and anatomical gyrus based on a standard brain atlas. The resulting channel-to-region mapping is summarized in the legend of Figure 2A.

A bandpass filter with cutoff frequencies of 0.01–0.20 Hz was employed to eliminate physiological noise such as respiration, cardiac activity, and low-frequency signal drift. Short-separation channels were not included in the current probe configuration. Global signal regression (GSR) was applied to reduce systemic physiological noise from scalp and superficial tissues. Channels with signal amplitude exceeding 2 SD from the grand mean were excluded to ensure data quality. The modified Beer–Lambert law was then used to convert optical densities into changes in oxy-hemoglobin (Oxy-Hb) and deoxy-hemoglobin (Deoxy-Hb) concentrations. Oxy-Hb was chosen as the primary indicator for subsequent analyses due to its superior signal-to-noise ratio compared to Deoxy-Hb (Strangman et al., 2002). VFT block waveforms were calculated with a block range of 0–125 s, a pre-baseline range of 0–10 s, and a post-baseline range of 70–125 s. A 60-s task period of phrase construction served as the time window for analyzing mean Oxy-Hb changes. Mean Oxy-Hb changes were calculated over the 0–60 s task period. The 60-s task window (0–60 s) was selected to capture sustained activation during verbal generation. Pre- and post-baseline windows were chosen to minimize signal drift and ensure stable baseline estimation. Block averaging was used instead of general linear model (GLM) analysis because it provides robust estimation of mean task-related activation and is less susceptible to motion artifacts commonly observed in pediatric fNIRS data. Linear fitting was applied between the two baselines. Based on the waveforms from all 19 channels, averaged waveforms of Oxy-Hb and Deoxy-Hb changes for all subjects in both groups were obtained.

Statistical analysis was conducted using SPSS version 27.0 (IBM Corp., NY, United States). Figures and graphs were generated using NirSpark software, Origin 2021, and Adobe Photoshop 2025. Data normality was assessed with the Shapiro–Wilk test. Demographic data was analyzed using independent samples t-tests or chi-square tests. Normality of all variables was first assessed via Shapiro–Wilk tests (detailed results in Supplementary Table S8). Shapiro–Wilk tests revealed non-normal distribution of Oxy-Hb signals in most channels. Consistent with our prior analytical plan addressing non-normal hemodynamic data, Mann–Whitney U nonparametric tests were uniformly applied for inter-group Oxy-Hb comparisons across all 19 channels, regardless of single-channel normality status. Independent samples t-tests were only used for normally distributed demographic variables, while chi-square tests were adopted for categorical demographic data. Accordingly, Spearman’s rank correlation was used for all correlation analyses between neural signals and behavioral/clinical indices (i.e., FSIQ and verbal production), which is appropriate for non-normal data.

For inter-channel functional connectivity analysis, we followed standard fNIRS analytical pipelines: Pearson correlation coefficients were computed to quantify pairwise temporal associations between Oxy-Hb signals. Although the raw hemodynamic data were non-normally distributed, Pearson correlation is widely accepted for evaluating linear coupling of continuous fNIRS time series in existing literature. Although frequency-domain metrics such as wavelet coherence are widely used to analyze oscillatory neural activities in fNIRS research, the present study focused on sustained static functional connectivity during the block-designed verbal fluency task. Therefore, we adopted time-domain correlation analysis to match our research objectives (Lin et al., 2024). All resulting correlation coefficients were then converted to Fisher’s z scores to stabilize variance. Group comparisons of connectivity strength were conducted using permutation tests, and all *p*-values were corrected via the false discovery rate (FDR) method (Andrade et al., 2024; Zhang et al., 2026).

### Control for multiple comparisons

**Figure 3:**
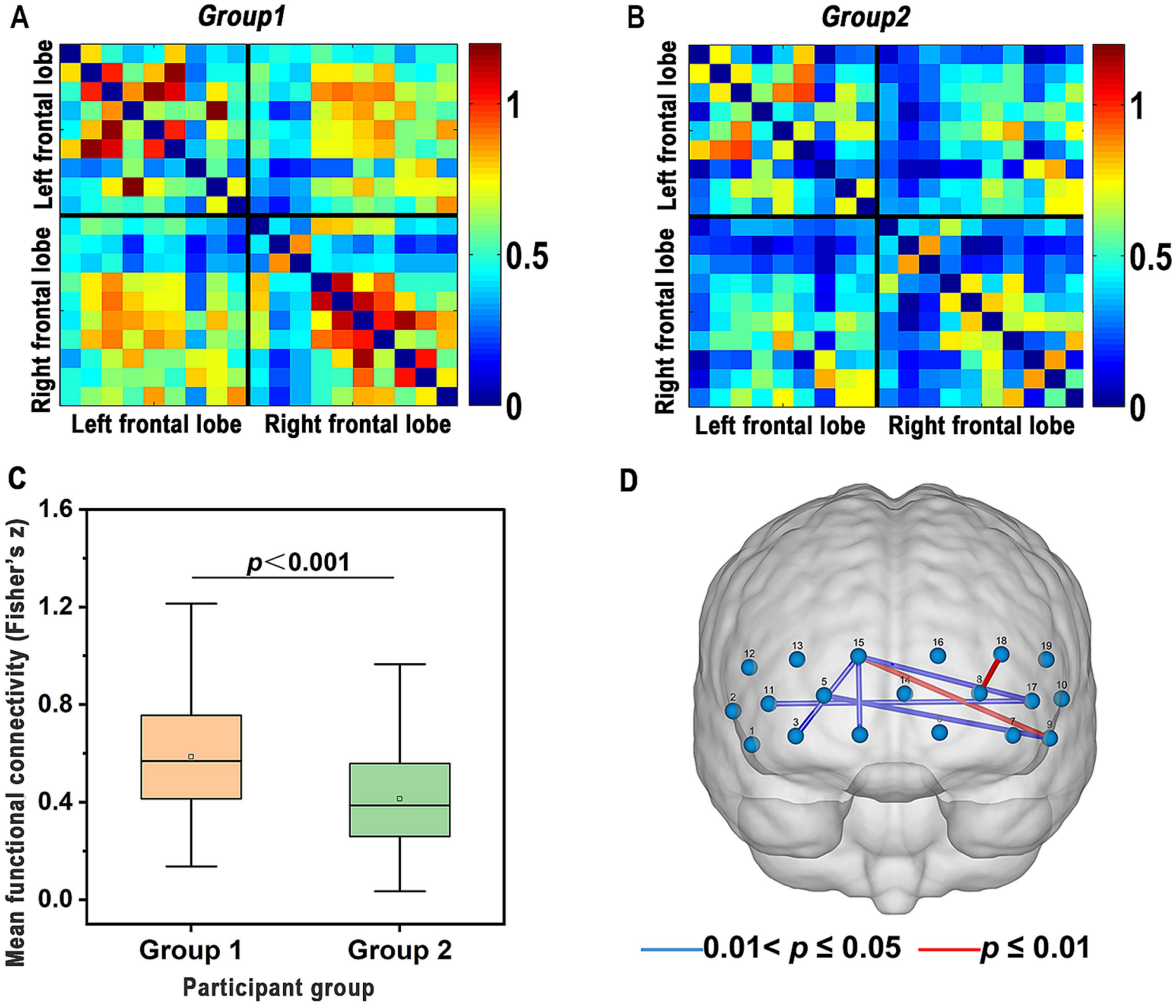
**(A,B)** Connectivity between the hemodynamic response of 19 channels. Left frontal lobe: Ch6-10 and 16–19; right frontal lobe: Ch1-5, 11–15. **(C)** Group 1 had a higher mean channel-to-channel connectivity strength than Group 2. **(D)** 7 channel pairs had significantly higher connectivity in Group 1 than Group 2 (*p* < 0.05). Group differences in functional connectivity were assessed using permutation tests with Benjamini–Hochberg FDR correction.

**Figure 4:**
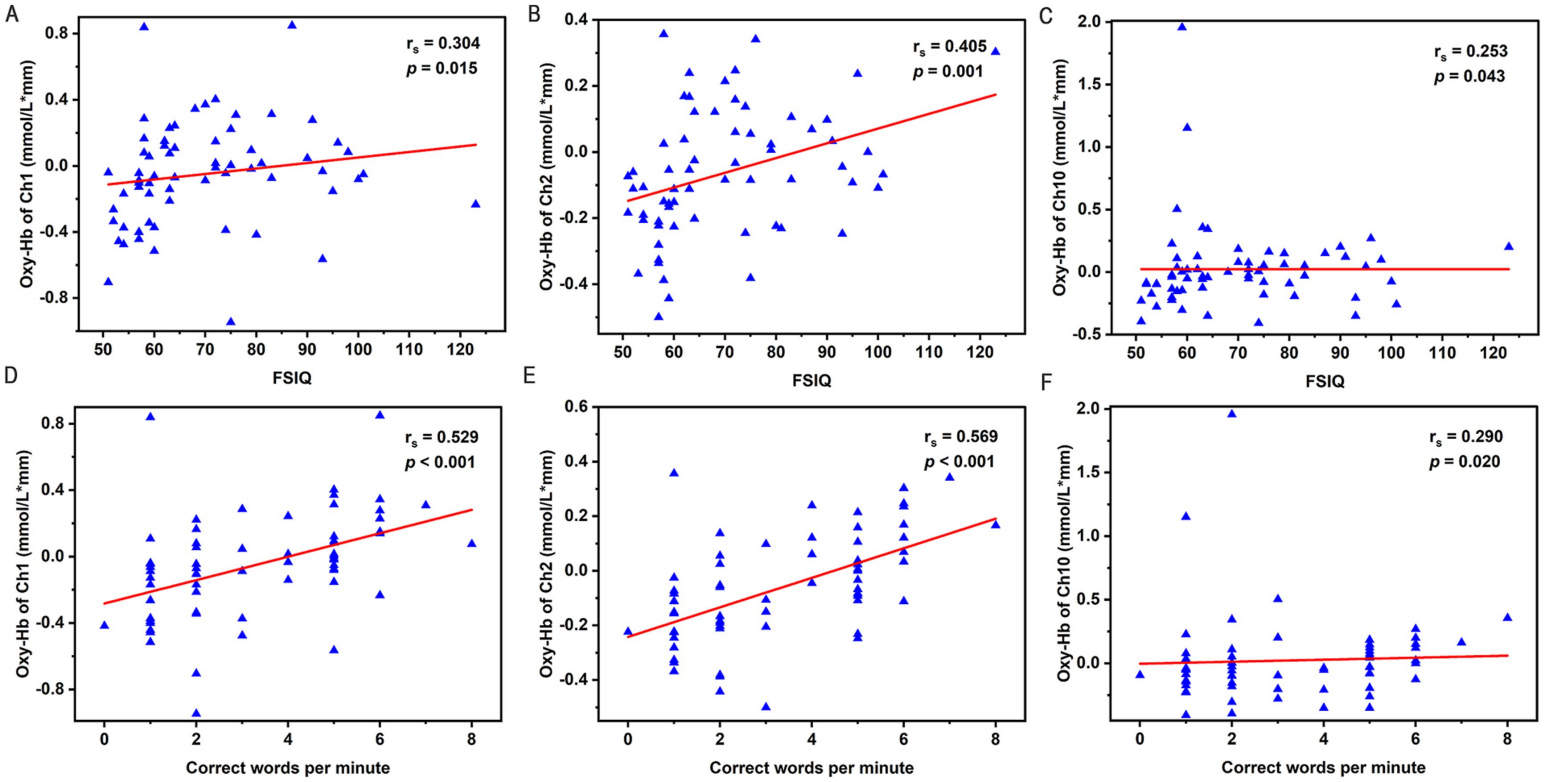
Correlation between FSIQ **(A–C)**, and correct words per minute **(D–F)** with the mean Oxy-Hb changes of Ch1, 2 and 10. Correlations were calculated using Spearman’s rank correlation.

To address the issue of multiple comparisons arising from the 19 fNIRS channels and their derived metrics, a Benjamini–Hochberg FDR correction was applied to the following families of statistical tests: (1) Channel-wise activation comparisons: Mann–Whitney U tests were uniformly adopted for Oxy-Hb intergroup comparisons across all 19 channels, with all uncorrected *p*-values adjusted via FDR (Supplementary Table S5). (2) Functional connectivity comparisons: Permutation tests were used to compare the connectivity strength for each unique channel-pair (171 pairs in total) between the two groups (Figure 3). (3) Correlation analyses: Spearman’s rank correlation coefficients calculated between the mean Oxy-Hb changes in all 19 channels and the clinical indices (FSIQ and Correct Words per Minute) (Figure 4).

The significance level was set at *p* < 0.05 (two-tailed) prior to correction. All *p*-values reported in the text, tables, and figures represent the Benjamini–Hochberg FDR-corrected values unless otherwise noted. The correction procedure was performed using the original, uncorrected *p*-values.

## Results

### Basic characteristics of the participants

Table 1 presents the basic characteristics of the participants who were grouped based on their FSIQ scores. The FSIQ ranges were 70–110 in Group 1 and 40–69 in Group 2. Group 1 (FSIQ ≥ 70) consisted of 29 males and 1 female, with a mean age of 8.70 years (*SD* = 2.38), while Group 2 (FSIQ < 70) included 33 males and 1 female, with a mean age of 7.85 years (*SD* = 1.78). There were no significant differences between the groups in terms of age (U = 387.500, *p* = 0.094), gender (chi-square = 0.008, *p* = 0.928), and parental care (chi-square = 0.302, *p* = 0.583). However, Group 1 showed significantly higher scores in the Full-Scale Intelligence Quotient (FSIQ) (*p* < 0.001). The mean scores for FSIQ in Group 1 were 82.10 (*SD* = 12.19). In contrast, Group 2 had mean scores of 51.88 (*SD* = 8.28), respectively.

**Table 1:** Demographic and clinical characteristics (mean ± SD).

	Group 1 (*n* = 30)	Group 2 (*n* = 34)	Mann–Whitney U/Chi-square value	*p*
Demographics				
Age (years)	8.70 ± 2.38	7.85 ± 1.78	387.500	0.094
Gender (male/female)	29/1	33/1	0.008	0.928
Parental care (yes/no)	23/7	24/10	0.302	0.583
Clinical characteristics				
Total score for CARS	33.77 ± 1.36	34.18 ± 1.11	619.500	0.128
Wechsler children’s intelligence test-IV				
Full-Scale Intelligence Quotient (FSIQ)	82.10 ± 12.19	51.88 ± 8.28	87.000	**< 0.001**
VFT behavioral performance				
Correct words/minute	5.23 ± 0.97	1.62 ± 0.74	2.000	**< 0.001**

FSIQ = Full-Scale Intelligence Quotient; VFT = Verbal Fluency Task; Oxy-Hb = oxygenated hemoglobin; fNIRS = functional near-infrared spectroscopy. *p*-values less than 0.05 are shown in bold.

### Verbal fluency task (VFT) behavioral performance

The behavioral performance on the VFT for both groups is summarized in Table 1. Group 1 (FSIQ ≥ 70) generated significantly more correct words per minute (mean = 5.23, *SD* = 0.97) compared to Group 2 (FSIQ < 70; mean = 1.62, *SD* = 0.74) (*U* = 2.000, *p* < 0.001). This indicates that, at the level of overt behavioral output, children with higher overall cognitive ability performed substantially better on the VFT.

### Mean Oxy-Hb changes during the VFT

A total of 19 NIRS channels were established to monitor changes in oxy-hemoglobin (Oxy-Hb) and deoxy-hemoglobin (Deoxy-Hb) in both groups during the VFT, with their locations illustrated in Figure 2A. Figure 2B display the grand average waveforms of Oxy-Hb and Deoxy-Hb for both groups. From an overall perspective, there were notable differences in the waveform patterns between the two groups. Specifically, in Group 1, the Oxy-Hb waveform across multiple channels typically exhibited a sharp peak with a regular slope during the task, followed by a return to baseline after task completion. In contrast, Group 2 generally exhibited Oxy-Hb waveforms with irregular slopes and less consistent peak patterns across many channels during the task (Figure 2B). Corresponding Deoxy-Hb waveforms were also obtained and exhibited typical task-related changes opposite Oxy-Hb. No obvious between-group differences were observed in the Deoxy-Hb time courses across channels. Data are presented as mean ± SD for both Oxy-Hb and Deoxy-Hb. Deoxy-Hb time courses are shown in Figure 2B, and detailed mean ± SD values are provided in Supplementary Table S4.

In further analysis, we compared mean task-related Oxy-Hb concentration changes across all 19 channels between the two groups. As presented in Table 2, Group 1 exhibited significantly greater Oxy-Hb activation than Group 2 in Channel 1 (*U* = 212.000, *Z* = −4.009, FDR-corrected *p* < 0.001) and Channel 2 (*U* = 160.000, *Z* = −4.709), FDR-corrected (*p* < 0.001). Consistent with our unified analytical framework, all channel-wise intergroup comparisons for both Oxy-Hb and Deoxy-Hb were performed using Mann–Whitney U nonparametric tests, with Benjamini–Hochberg FDR correction applied to control for multiple testing bias. These activation patterns align with the grand-averaged hemodynamic waveforms displayed in Figure 2B. We further conducted group comparisons of Deoxy-Hb signals across all channels. Most channels showed no significant intergroup differences after FDR adjustment (all FDR-corrected *p* > 0.05). Significant group disparities in Deoxy-Hb responses were only observed in Channel 3 (*U* = 665, *Z* = 2.085, FDR-corrected *p* < 0.05) and Channel 10 (*U* = 671, *Z* = 2.166, FDR-corrected *p* < 0.05). Complete statistical results for Deoxy-Hb are provided in Supplementary Table S5.

**Table 2:** Between-group comparisons of brain activation (Oxy-Hb) in channel 1 and channel 2 using Mann–Whitney U.

Channel	S-D	Group 1	Group 2	Mann–Whitney U	Z	*p* (FDR corrected)
1	S2-D2	0.0955	−0.1769	212.000	**−4.009**	**< 0.001**
2	S2-D7	0.0548	−0.1668	160.000	**−4.709**	**< 0.001**

Between-group comparisons of Oxy-Hb signals were conducted using Mann–Whitney U tests, with FDR correction applied to control for multiple comparisons. Group mean values are presented solely for descriptive reference. Bold values denote statistically significant intergroup differences (FDR-corrected *p* < 0.05). *p*-values less than 0.05 are shown in bold.

### Functional connectivity

Following the calculation of functional connectivity, two 19 × 19 correlation matrices were generated for Groups 1 and 2 (Figures 4A,B; Supplementary Tables S6, S7). Notably, Group 1 exhibited significantly stronger functional connectivity compared to Group 2 (permutation statistic = 4.82, FDR-corrected *p* < 0.001), with mean channel-to-channel connectivity strengths of 0.586 (*SD* = 0.521) and 0.413 (*SD* = 0.508), respectively (Figure 3C). Specifically, compared to Group 2, Group 1 demonstrated significantly higher connectivity strength in 7 pairwise channel-to-channel comparisons (permutation statistics ranged from 3.46 to 4.47, all FDR-corrected *p* < 0.05) (Table 3; Figure 3D). All group comparisons of functional connectivity were performed on Fisher’s z-transformed values using permutation tests, and all *p*-values were corrected using the false discovery rate (FDR) method. Among these channel pairs, Channel 15 appeared most frequently, occurring 4 times.

**Table 3:** Channel pairs with significant differences in connectivity strength between groups (mean ± SD).

Channel to channel	Connectivity strength		Permutation statistic	p (FDR corrected)
	Group 1	Group 2		
3 ~ 15	0.58 ± 0.32	0.23 ± 0.51	3.38	**0.0357**
4 ~ 15	0.51 ± 0.34	0.12 ± 0.48	3.80	**0.0191**
5 ~ 9	0.65 ± 0.28	0.39 ± 0.34	3.40	**0.0357**
8 ~ 18	0.84 ± 0.18	0.57 ± 0.30	4.26	**0.0061**
9 ~ 15	0.53 ± 0.39	0.08 ± 0.40	4.31	**0.0061**
11 ~ 17	0.71 ± 0.24	0.42 ± 0.36	3.40	**0.0357**
15 ~ 17	0.58 ± 0.37	0.23 ± 0.46	3.33	**0.0357**

Raw Pearson correlation coefficients (mean ± SD) are presented for interpretability. Group comparisons were conducted on Fisher’s z-transformed values via permutation tests, and *p*-values were corrected using the false discovery rate (FDR) method. *p*-values less than 0.05 are shown in bold.

### Correlations with clinical characteristics

We next evaluated the correlation between FSIQ and the mean Oxy-Hb and Deoxy-Hb changes across all channels using Spearman’s rank correlation (Table 4; Supplementary Table S9). Significant positive correlations were observed between FSIQ and Oxy-Hb changes in Channels 1 (*r_s_* = 0.304, *p* < 0.05), 2 (*r_s_* = 0.405, *p* < 0.05) and 10 (*r_s_* = 0.253, *p* < 0.05) (Figure 4; Supplementary Figure S2). In addition to the significant positive correlation between FSIQ and oxy-Hb activation in Ch1, significant negative correlations were also found between FSIQ and deoxy-Hb activation in multiple channels, including Ch3 (*r_s_* = −0.258, *p* < 0.05), Ch7 (*r_s_* = −0.358, *p* < 0.05), and Ch10 (*r_s_* = −0.247, *p* < 0.05). These negative correlations suggest that higher IQ was associated with greater task-induced reduction in deoxy-Hb, which is consistent with a more typical neurovascular coupling pattern. Scatter plots of these correlations are provided in Supplementary Figure S2. Activation in channels 1 (*r_s_* = 0.405, *p* < 0.05), 2 (*r_s_* = 0.405, *p* < 0.05), and 10 (*r_s_* = 0.290, *p* < 0.05) were also significantly correlated with verbal production (Correct Words per minute) (Table 4; Figure 4). Notably, only Channels 1, 2, and 10 were significantly associated with both FSIQ and verbal production; all other channels showed no significant concurrent correlations with these two measures.

**Table 4:** Significant Spearman correlations of core Oxy-Hb channels.

Variables	FSIQ (*r_s_*)	*p*	Correct words (*r_s_*)	*p*
Oxy-Hb Ch1	0.304	**0.015**	0.529	**< 0.001**
Oxy-Hb Ch2	0.405	**0.001**	0.569	**< 0.001**
Oxy-Hb Ch10	0.253	**0.043**	0.290	**0.020**

Spearman rank correlation (*r_s_*) was used for all analyses due to non-normal data distribution. FSIQ, full-scale intelligence quotient. *p*-values less than 0.05 are shown in bold.

## Discussion

In this study, we used fNIRS to explore prefrontal activation and connectivity in ASD children with different levels of overall cognitive ability (FSIQ) during a VFT. Our findings reveal that differences in cognitive resource availability are associated with distinct patterns of neural engagement during this complex verbal-executive task. Since the lexical properties of the task materials were strictly controlled, the observed inter-group differences in neural activation are more likely to reflect differences in cognitive processing rather than uneven difficulty in lexical access or word-level processing.

We found lower activation in the inferior frontal gyrus (IFG, encompassing Broca’s area) in the lower-FSIQ group. The IFG is pivotal for lexical retrieval and selection as well as response inhibition (George et al., 2025; Gertel et al., 2020). The observed hypoactivation in this region may therefore reflect less efficient search and retrieval of words from the mental lexicon, and/or weaker suppression of task-irrelevant responses during the VFT in children with lower cognitive resources. Stronger activation in the higher FSIQ group may reflect more efficient cognitive resource recruitment, whereas weaker activation in the lower FSIQ group may indicate limited cognitive capacity or relatively greater task difficulty. Lower Oxy-Hb in Group 2 may reflect insufficient task-related neural recruitment, interference from the default mode network, or inefficient neural resource allocation under cognitive demand. Hypoactivation of the inferior frontal gyrus is closely associated with impaired lexical retrieval and syntactic organization, which further contributes to poor verbal performance in children with lower intellectual level (Alvarez-Rivero et al., 2026; Nasiri et al., 2022). Critically, our correlation analyses extend this finding by demonstrating that the level of IFG (Channels 1 and 2) activation was positively associated not only with general cognitive capacity (FSIQ) but also with the quantitative verbal output (Correct Words per minute). This dual association underscores the IFG’s central role as a neural interface where domain-general cognitive resources converge with language-specific processes to support fluent verbal production. Our results align with and extend previous research implicating the IFG in language and executive function challenges with ASD (Deng et al., 2023; Su et al., 2023; Su et al., 2020; Su et al., 2021).

Functional connectivity analysis further supported these results. Significantly, ASD children with an FSIQ <70 were observed to have weaker mean global connectivity than those with FSIQ ≥70, indicating a more severely impaired integration of neural networks critical for verbal fluency and cognitive processing. This widespread reduction in connectivity was particularly evident in seven specific channel pairs that showed significantly lower strength in the lower-FSIQ group (Table 3; Figure 3D). This pattern of multifocal underconnectivity, especially prominent in the autism group with lower cognitive ability, provides evidence from within the spectrum supporting the “underconnectivity” hypothesis in autism. Future research could explore whether neuromodulation technologies can enhance these specific connections. Such studies may open new pathways to improve cognitive abilities in autistic children with lower cognitive capacity (Manard et al., 2017; Ohtani et al., 2021; Wan et al., 2024; Zhang and Roeyers, 2019).

Further analysis revealed that the reduction in functional connectivity was not uniformly distributed but concentrated in specific channel pairs (Table 3; Figure 3D). Notably, Channel 15 (corresponding to the frontopolar cortex, FPC) was implicated in multiple connections showing significant group differences (3–15, 4–15, 9–15, 15–17). The FPC is often regarded as a high-level cognitive hub, responsible for maintaining internal goals, switching between task sets, and directing attention to behaviorally relevant information (Hui et al., 2024; Mou et al., 2025). Its coordinated interaction with the dorsolateral prefrontal cortex (DLPFC—involved in maintaining and monitoring external goals) and the ventrolateral prefrontal cortex/inferior frontal gyrus (IFG—involved in response selection and inhibition) is crucial for performing a task like the VFT, which requires continuous generation, monitoring, and switching of semantic search strategies. The significantly weakened connectivity involving the FPC in the lower-FSIQ group suggests a specific deficit in cognitive flexibility and in the ability to coordinate resources across different prefrontal sub-regions during complex tasks. This “intra-prefrontal network discoordination” may partly explain their poorer performance on verbal fluency tasks that demand dynamic cognitive control.

The positive correlation between FSIQ and activation, verbal production and activation, in channels 1, 2 (IFG), and 10 (PT) reinforces the link between general cognitive capacity and prefrontal recruitment during verbal-executive processing. Neuroanatomically, the pars triangularis (PT, Brodmann area 45) is key for lexical retrieval, semantic selection, and implementing cognitive control over verbal output. Our finding that PT (Channel 10) activation was correlated with both higher FSIQ and better verbal output underscores its integral role in supporting executive control processes coupled with lexical-semantic processing during verbal tasks. Given the absence of non-verbal executive control tasks (e.g., Stroop, Go/NoGo) in the present paradigm, we cannot definitively distinguish language-selective versus domain-general executive engagement within this region. Thus, the coordinated engagement of the inferior frontal gyrus (IFG) and its pars triangularis (PT)-supporting lexical-semantic processing and executive regulation of verbal output, respectively—appears to be a key neural signature of efficient verbal-executive integration in children with higher cognitive ability. In addition to the Oxy-Hb findings, significant negative correlations between FSIQ and Deoxy-Hb activation in multiple channels further support this interpretation. Higher IQ was associated with a stronger reduction in Deoxy-Hb during the task, which, together with the increased Oxy-Hb response, suggests more efficient and typical task-related brain activation in children with higher intellectual function. This pattern aligns with previous fNIRS studies showing that better cognitive performance is linked to clearer task-evoked hemoglobin changes, reflecting more effective neurovascular coupling.

It should be noted that interpretations of neural activation patterns require caution. While the weaker or atypical hemodynamic responses observed in several prefrontal channels in the lower-FSIQ group may indeed reflect inefficient recruitment of neural resources in relevant regions, an alternative explanation exists. This group may rely on a qualitatively different cognitive or neural strategy when confronting the VFT challenge. For instance, they might depend more heavily on rote retrieval from memory, engage in more diffuse semantic associations, or recruit resources from brain regions not covered by the present fNIRS probe array—such as temporal lobe semantic stores or subcortical structures. Future studies employing whole-brain imaging techniques could help unveil such potential strategic compensation or divergence.

In summary, this study elucidates neurofunctional correlations of verbal fluency in ASD children with varying cognitive abilities. The IFG emerges as a potential neural marker for language capacity, with its activation level predicting both cognitive resource availability and verbal output. Furthermore, the coordinated engagement of the IFG and PT (pars triangularis)—subserving lexical-semantic and verbal-executive control processes, respectively—appears crucial for efficient verbal-executive integration. These findings support the utility of fNIRS as a practical tool for assessing language-executive profiles in ASD (Whiteside et al., 2016) and may guide the development of more personalized interventions that target specific prefrontal subsystems.

### Limitations and future directions

Several limitations should be acknowledged. First, probe positions were placed according to the international 10–20 system without 3D digitization. Minor individual differences in probe placement may exist, although standardized positioning minimized variability. In addition, the probe configuration did not include short-separation channels, which may limit the correction of superficial systemic noise, representing a potential limitation of the present fNIRS setup. Second, the current study lacks an age-matched neurotypical control group, which weakens the interpretability of our findings. We can only compare neural patterns between two ASD subgroups rather than distinguish autism-specific prefrontal activation alterations from typical developmental responses during verbal fluency tasks. Future studies should recruit a cohort of neurotypical children to address this limitation. Third, the sample size was moderate, and the cross-sectional design precludes causal or developmental inferences. Fourth, while fNIRS offers excellent temporal resolution and motion tolerance, its spatial resolution is limited compared with fMRI. Additionally, our fNIRS probe array was restricted to the prefrontal cortex; thus, we could not assess the potential contributions of other brain regions implicated in language and executive function, such as the temporal and parietal lobes. Future studies could combine fNIRS with structural MRI for more precise anatomical localization or use whole-head fNIRS systems for broader coverage. Fifth, the VFT variant used in this study—phrase construction based on Chinese characters—differs from classic letter or category fluency tasks. While it effectively probes verbal-executive integration, the additional work-formation component may recruit distinct neural resources. Cross-cultural task comparisons are warranted. Sixth, the high male-to-female ratio in our sample (62/64) limits the generalizability of our findings to females with ASD. Given emerging evidence of sex-specific neural phenotypes in ASD, future studies should strive to recruit more balanced cohorts to examine potential sex differences in prefrontal activation and connectivity during verbal-executive tasks.

Future research should employ longitudinal designs to track the evolution of these neural patterns and examine their predictive value for language development. Intervention studies could test whether modulating identified prefrontal networks (e.g., via neurofeedback or cognitive training) improves verbal fluency. Finally, multimodal imaging (e.g., fNIRS-EEG) could provide a more comprehensive understanding of the neurodynamics underlying verbal-executive integration in ASD.

## Conclusion

In conclusion, this fNIRS study revealed distinct neurofunctional profiles associated with verbal fluency in children with ASD stratified by cognitive ability. Compared to those with higher FSIQ (≥70), children with lower FSIQ exhibited significantly lower activation and compromised functional connectivity within prefrontal regions critical for language processing—specifically the inferior frontal gyrus (supporting lexical-syntactic integration) and the dorsolateral prefrontal cortex (involved in executive regulation). These results suggest that verbal fluency challenges with ASD may stem from inefficient neural coordination within prefrontal cognitive-linguistic networks, encompassing both the inferior frontal gyrus (for lexical-syntactic processes) and the dorsolateral prefrontal cortex (for executive regulation). The findings provide a neurofunctional framework for understanding individual differences in ASD and may inform the development of biomarker-guided, mechanism-based interventions.

## Data Availability

The raw data supporting the conclusions of this article will be made available by the authors, without undue reservation.

## Supplementary material

SUPPLEMENTARY FIGURE S1 **(A)** Mean Oxy-Hb changes across all channels in Group 1. **(B)** Mean Oxy-Hb changes across all channels in Group 2. **(C,D)** Channel-wise *p*-values for intergroup activation differences derived from independent samples *t*-tests in the preliminary analysis. This figure was produced via parametric statistical analysis and serves solely as a supplementary reference. All definitive statistical conclusions presented in the main manuscript are based on uniform Mann–Whitney U nonparametric tests with Benjamini–Hochberg FDR correction applied to all 19 channels.

SUPPLEMENTARY FIGURE S2 Spearman correlations between Deoxy-Hb signals of Channel 3 **(A)**, Channel 7 **(B)**, Channel 10 **(C)** and FSIQ. Corresponding Spearman correlation coefficients (*r_s_*) and *p*-values are annotated within each subplot.
