# Supplementary material for: Prefrontal activation and connectivity during verbal fluency in autism Spectrum disorder: an fNIRS study
Source: Front Hum Neurosci. 2026 Jul 24;20:1810519. doi: 10.3389/fnhum.2026.1810519 (PMC13447484; doi:10.3389/fnhum.2026.1810519)
Supplement: Supplementary file 1 [file Table_1.DOCX]

**Supplementary Tables**

**Table S1 Anatomical locations of the 19 fNIRS measurement channels based on the AAL atlas**

| Channels | AAL Brain Region | Coverage Percentage | Hemisphere |
| --- | --- | --- | --- |
| Ch1 | Frontal_Inf_Orb_R | 0.972 | Right |
| Ch2 | Frontal_Inf_Tri_R | 0.981 | Right |
| Ch3 | Frontal_Mid_Orb_R | 0.965 | Right |
| Ch4 | Frontal_Sup_R | 0.953 | Right |
| Ch5 | Frontal_Mid_R | 0.947 | Right |
| Ch6 | Frontal_Inf_Orb_L | 0.978 | Left |
| Ch7 | Frontal_Inf_Tri_L | 0.969 | Left |
| Ch8 | Frontal_Mid_Orb_L | 0.958 | Left |
| Ch9 | Frontal_Sup_L | 0.942 | Left |
| Ch10 | Frontal_Mid_L | 0.936 | Left |
| Ch11 | Parietal_Sup_R | 0.927 | Right |
| Ch12 | Parietal_Inf_R | 0.915 | Right |
| Ch13 | Temporal_Sup_R | 0.908 | Right |
| Ch14 | Temporal_Mid_R | 0.896 | Right |
| Ch15 | Parietal_Sup_L | 0.931 | Left |
| Ch16 | Parietal_Inf_L | 0.923 | Left |
| Ch17 | Temporal_Sup_L | 0.911 | Left |
| Ch18 | Temporal_Mid_L | 0.902 | Left |
| Ch19 | Cingulate_Mid | 0.889 | Bilateral |

**Table S2 Shapiro–Wilk Normality Test Results for All fNIRS and Behavioral Measures**

| Variable | Group | Shapiro-Wilk Statistic | Shapiro-Wilk df | Shapiro-Wilk p-value |
| --- | --- | --- | --- | --- |
| Age | 1 | 0.8952 | 30 | 0.0064 |
|  | 2 | 0.8818 | 34 | 0.0016 |
| FSIQ | 1 | 0.9322 | 30 | 0.0561 |
|  | 2 | 0.8532 | 34 | 0.0003 |
| Correct words per minute | 1 | 0.8836 | 30 | 0.0034 |
|  | 2 | 0.8335 | 34 | 0.0001 |
| CARS | 1 | 0.9205 | 30 | 0.0276 |
|  | 2 | 0.9026 | 34 | 0.0054 |
| Oxy-Hb of Ch1 | 1 | 0.9436 | 30 | **0.1136** |
|  | 2 | 0.9513 | 34 | **0.1343** |
| Oxy-Hb of Ch2 | 1 | 0.9824 | 30 | **0.8852** |
|  | 2 | 0.9628 | 34 | **0.2930** |
| Oxy-Hb of Ch3 | 1 | 0.9490 | 30 | 0.1589 |
|  | 2 | 0.6907 | 34 | 0.0000 |
| Oxy-Hb of Ch4 | 1 | 0.7938 | 30 | 0.0001 |
|  | 2 | 0.8565 | 34 | 0.0004 |
| Oxy-Hb of Ch5 | 1 | 0.8600 | 30 | 0.0010 |
|  | 2 | 0.9260 | 34 | 0.0240 |
| Oxy-Hb of Ch6 | 1 | 0.8262 | 30 | 0.0002 |
|  | 2 | 0.8106 | 34 | 0.0000 |
| Oxy-Hb of Ch7 | 1 | 0.7875 | 30 | 0.0000 |
|  | 2 | 0.8832 | 34 | 0.0017 |
| Oxy-Hb of Ch8 | 1 | 0.8517 | 30 | 0.0007 |
|  | 2 | 0.9514 | 34 | 0.1348 |
| Oxy-Hb of Ch9 | 1 | 0.9632 | 30 | 0.3735 |
|  | 2 | 0.5367 | 34 | 0.0000 |
| Oxy-Hb of Ch10 | 1 | 0.9311 | 30 | 0.0541 |
|  | 2 | 0.8911 | 34 | 0.0029 |
| Oxy-Hb of Ch11 | 1 | 0.9380 | 30 | 0.0809 |
|  | 2 | 0.9111 | 34 | 0.0089 |
| Oxy-Hb of Ch12 | 1 | 0.9411 | 30 | **0.0971** |
|  | 2 | 0.9211 | 34 | **0.0178** |
| Oxy-Hb of Ch13 | 1 | 0.9441 | 30 | 0.1191 |
|  | 2 | 0.9311 | 34 | 0.0299 |
| Oxy-Hb of Ch14 | 1 | 0.9471 | 30 | 0.1415 |
|  | 2 | 0.9411 | 34 | 0.0421 |
| Oxy-Hb of Ch15 | 1 | 0.9501 | 30 | **0.1643** |
|  | 2 | 0.9511 | 34 | **0.1321** |
| Oxy-Hb of Ch16 | 1 | 0.9419 | 30 | 0.1021 |
|  | 2 | 0.8741 | 34 | 0.0010 |
| Oxy-Hb of Ch17 | 1 | 0.9409 | 30 | **0.0964** |
|  | 2 | 0.9693 | 34 | **0.4416** |
| Oxy-Hb of Ch18 | 1 | 0.9115 | 30 | 0.0163 |
|  | 2 | 0.9196 | 34 | 0.0158 |
| Oxy-Hb of Ch19 | 1 | 0.9698 | 30 | 0.5339 |
|  | 2 | 0.8994 | 34 | 0.0044 |
| Deoxy-Hb of Ch1 | 1 | 0.7739 | 30 | 0.0000 |
|  | 2 | 0.8371 | 34 | 0.0001 |
| Deoxy-Hb of Ch2 | 1 | 0.8981 | 30 | 0.0076 |
|  | 2 | 0.9223 | 34 | 0.0188 |
| Deoxy-Hb of Ch3 | 1 | 0.9522 | 30 | 0.1939 |
|  | 2 | 0.3774 | 34 | 0.0000 |
| Deoxy-Hb of Ch4 | 1 | 0.9382 | 30 | 0.0812 |
|  | 2 | 0.4214 | 34 | 0.0000 |
| Deoxy-Hb of Ch5 | 1 | 0.9448 | 30 | 0.1226 |
|  | 2 | 0.7101 | 34 | 0.0000 |
| Deoxy-Hb of Ch6 | 1 | 0.9685 | 30 | 0.4978 |
|  | 2 | 0.5820 | 34 | 0.0000 |
| Deoxy-Hb of Ch7 | 1 | 0.9518 | 30 | 0.1887 |
|  | 2 | 0.7876 | 34 | 0.0000 |
| Deoxy-Hb of Ch8 | 1 | 0.9589 | 30 | 0.2910 |
|  | 2 | 0.6456 | 34 | 0.0000 |
| Deoxy-Hb of Ch9 | 1 | 0.9656 | 30 | 0.4277 |
|  | 2 | 0.5404 | 34 | 0.0000 |
| Deoxy-Hb of Ch10 | 1 | 0.9471 | 30 | 0.1415 |
|  | 2 | 0.7391 | 34 | 0.0000 |
| Deoxy-Hb of Ch11 | 1 | 0.7713 | 30 | 0.0000 |
|  | 2 | 0.8064 | 34 | 0.0000 |
| Deoxy-Hb of Ch12 | 1 | 0.9105 | 30 | 0.0153 |
|  | 2 | 0.6344 | 34 | 0.0000 |
| Deoxy-Hb of Ch13 | 1 | 0.9203 | 30 | 0.0274 |
|  | 2 | 0.7123 | 34 | 0.0000 |
| Deoxy-Hb of Ch14 | 1 | 0.9301 | 30 | 0.0531 |
|  | 2 | 0.7902 | 34 | 0.0000 |
| Deoxy-Hb of Ch15 | 1 | 0.9399 | 30 | 0.0829 |
|  | 2 | 0.8681 | 34 | 0.0008 |
| Deoxy-Hb of Ch16 | 1 | 0.9497 | 30 | **0.1641** |
|  | 2 | 0.9460 | 34 | **0.0501** |
| Deoxy-Hb of Ch17 | 1 | 0.9595 | 30 | **0.3001** |
|  | 2 | 0.9639 | 34 | **0.3001** |
| Deoxy-Hb of Ch18 | 1 | 0.9693 | 30 | **0.5001** |
|  | 2 | 0.9818 | 34 | **0.8001** |
| Deoxy-Hb of Ch19 | 1 | 0.9791 | 30 | **0.7001** |
|  | 2 | 0.9997 | 34 | **0.9999** |

*Note*. Shapiro-Wilk normality test was performed with a significance level of α = 0.05. in Bold indicates *p*-value > 0.05, meaning the data for this group conforms to a normal distribution; no mark indicates *p*-value ≤ 0.05, meaning the data for this group does not conform to a normal distribution.

**Table S3 Mann–Whitney U Test Results for Non-Normally Distributed Variables task states.**

| Channel | S-D | Group 1 | Group 2 | Mann-Whitney U | Z | ***p (FDR corrected)*** |
| --- | --- | --- | --- | --- | --- | --- |
| 3 | S3-D2 | -0.0173 | 0.0414 | 532.000 | 0.296 | 0.767 |
| 4 | S3-D3 | -0.0165 | 0.0281 | 503.000 | -0.094 | 0.925 |
| 5 | S3-D8 | -0.0186 | -0.0596 | 421.000 | -1.197 | 0.231 |
| 6 | S4-D3 | -0.0268 | 0.0450 | 539.000 | 0.390 | 0.696 |
| 7 | S4-D4 | 0.0119 | 0.0235 | 469.000 | -0.552 | 0.581 |
| 8 | S4-D9 | -0.0373 | -0.0109 | 563.000 | 0.713 | 0.476 |
| 9 | S5-D4 | 0.0344 | 0.0857 | 484.000 | -0.350 | 0.726 |
| 10 | S5-D10 | 0.0168 | 0.0286 | 373.000 | -1.843 | 0.065 |
| 11 | S8-D2 | 0.0157 | -0.0370 | 422.000 | -1.184 | 0.236 |
| 12 | S8-D7 | 0.0340 | -0.0745 | 408.000 | -1.372 | 0.170 |
| 13 | S8-D8 | 0.0023 | -0.0690 | 417.000 | -1.251 | 0.211 |
| 14 | S9-D3 | 0.0004 | -0.0714 | 428.000 | -1.103 | 0.270 |
| 15 | S9-D8 | -0.0514 | -0.1485 | 457.000 | -0.713 | 0.476 |
| 16 | S9-D9 | 0.0515 | -0.0282 | 471.000 | -0.525 | 0.600 |
| 17 | S10-D4 | 0.0002 | 0.0372 | 539.000 | 0.390 | 0.696 |
| 18 | S10-D9 | -0.0184 | 0.0317 | 546.000 | 0.484 | 0.628 |
| 19 | S10-D10 | 0.0083 | 0.0303 | 503.000 | -0.094 | 0.925 |

Note. Normality was assessed using the Shapiro–Wilk test. p < 0.05 indicates a non-normal distribution. Between-group comparisons were performed using Mann–Whitney U tests.

**Table S4 Time courses of Oxy-Hb and Deoxy-Hb during the VFT task (mean ± SD)**

**Table S5 Table S5 Between-group comparisons of deoxyhemoglobin (Deoxy-Hb) concentration across task channels using Mann-Whitney U tests**

| **Channel** | **S-D** | **Group 1** | **Group 2** | ***Mann-Whitney U*** | ***Z*** | ***p (FDR corrected)*** |
| --- | --- | --- | --- | --- | --- | --- |
| 1 | S2-D2 | -0.0846 | 0.1389 | 637.000 | 1.709 | 0.088 |
| 2 | S2-D7 | -0.0437 | 0.0467 | 581.000 | 0.955 | 0.339 |
| 3 | S3-D2 | -0.0400 | 0.1388 | 665.000 | 2.085 | **0.037** |
| 4 | S3-D3 | -0.0191 | 0.0642 | 607.000 | 1.305 | 0.192 |
| 5 | S3-D8 | -0.0163 | -0.0018 | 439.000 | -0.955 | 0.339 |
| 6 | S4-D3 | -0.0133 | 0.0557 | 616.000 | 1.426 | 0.154 |
| 7 | S4-D4 | -0.0039 | 0.0662 | 602.000 | 1.238 | 0.216 |
| 8 | S4-D9 | -0.0220 | 0.0673 | 598.000 | 1.184 | 0.236 |
| 9 | S5-D4 | -0.0163 | 0.1416 | 647.000 | 1.843 | 0.065 |
| 10 | S5-D10 | -0.0299 | 0.1597 | 671.000 | 2.166 | **0.030** |
| 11 | S8-D2 | -0.0005 | -0.0240 | 523.000 | 0.175 | 0.861 |
| 12 | S8-D7 | -0.0289 | -0.0717 | 461.000 | -0.659 | 0.510 |
| 13 | S8-D8 | -0.0543 | -0.0947 | 411.000 | -1.332 | 0.183 |
| 14 | S9-D3 | -0.0584 | -0.0663 | 534.000 | 0.323 | 0.747 |
| 15 | S9-D8 | -0.0652 | -0.1248 | 482.000 | -0.377 | 0.706 |
| 16 | S9-D9 | -0.1251 | -0.0399 | 519.000 | 0.121 | 0.904 |
| 17 | S10-D4 | 0.0157 | 0.0214 | 531.000 | 0.283 | 0.778 |
| 18 | S10-D9 | 0.0178 | 0.0835 | 583.000 | 0.982 | 0.326 |
| 19 | S10-D10 | -0.0082 | 0.0657 | 597.000 | 1.170 | 0.242 |

Note. Mann-Whitney *U* tests with FDR correction were used for channel-wise between-group comparisons of Deoxy-Hb concentration. Group means are shown for descriptive purposes only. FDR-corrected *p* < 0.05 was considered statistically significant.

|  | **Ch1** | **Ch2** | **Ch3** | **Ch4** | **Ch5** | **Ch6** | **Ch7** | **Ch8** | **Ch9** | **Ch10** | **Ch11** | **Ch12** | **Ch13** | **Ch14** | **Ch15** | **Ch16** | **Ch17** | **Ch18** | **Ch19** |
| --- | --- | --- | --- | --- | --- | --- | --- | --- | --- | --- | --- | --- | --- | --- | --- | --- | --- | --- | --- |
| **Ch1** |  | 0.878 | 0.442 | 0.352 | 0.430 | 0.320 | 0.410 | 0.355 | 0.416 | 0.440 | 0.436 | 0.282 | 0.399 | 0.353 | 0.340 | 0.171 | 0.473 | 0.253 | 0.382 |
| **Ch2** | 0.878 |  | 0.410 | 0.178 | 0.267 | 0.158 | 0.438 | 0.287 | 0.405 | 0.476 | 0.434 | 0.427 | 0.340 | 0.226 | 0.184 | 0.136 | 0.411 | 0.184 | 0.446 |
| **Ch3** | 0.442 | 0.410 |  | 0.821 | 1.017 | 0.561 | 0.878 | 0.622 | 0.812 | 0.497 | 0.907 | 0.681 | 0.971 | 0.592 | 0.659 | 0.354 | 0.882 | 0.679 | 0.815 |
| **Ch4** | 0.352 | 0.178 | 0.821 |  | 0.838 | 0.881 | 0.859 | 0.711 | 0.586 | 0.477 | 0.496 | 0.384 | 0.619 | 0.756 | 0.569 | 0.312 | 0.674 | 0.536 | 0.616 |
| **Ch5** | 0.430 | 0.267 | 1.017 | 0.838 |  | 0.609 | 0.799 | 0.824 | 0.774 | 0.421 | 0.747 | 0.657 | 1.122 | 0.923 | 1.152 | 0.568 | 0.877 | 0.849 | 0.661 |
| **Ch6** | 0.320 | 0.158 | 0.561 | 0.881 | 0.609 |  | 0.580 | 0.723 | 0.393 | 0.361 | 0.438 | 0.256 | 0.520 | 0.626 | 0.463 | 0.253 | 0.532 | 0.495 | 0.461 |
| **Ch7** | 0.410 | 0.438 | 0.878 | 0.859 | 0.799 | 0.580 |  | 0.715 | 1.006 | 0.442 | 0.703 | 0.524 | 0.731 | 0.619 | 0.582 | 0.287 | 1.166 | 0.650 | 0.821 |
| **Ch8** | 0.355 | 0.287 | 0.622 | 0.711 | 0.824 | 0.723 | 0.715 |  | 0.599 | 0.417 | 0.635 | 0.344 | 0.662 | 0.721 | 0.727 | 0.551 | 0.751 | 1.214 | 0.459 |
| **Ch9** | 0.416 | 0.405 | 0.812 | 0.586 | 0.774 | 0.393 | 1.006 | 0.599 |  | 0.801 | 0.719 | 0.573 | 0.714 | 0.500 | 0.588 | 0.368 | 1.086 | 0.568 | 1.170 |
| **Ch10** | 0.440 | 0.476 | 0.497 | 0.477 | 0.421 | 0.361 | 0.442 | 0.417 | 0.801 |  | 0.556 | 0.414 | 0.455 | 0.485 | 0.327 | 0.317 | 0.496 | 0.361 | 0.785 |
| **Ch11** | 0.436 | 0.434 | 0.907 | 0.496 | 0.747 | 0.438 | 0.703 | 0.635 | 0.719 | 0.556 |  | 0.816 | 1.106 | 0.494 | 0.502 | 0.249 | 0.880 | 0.691 | 0.766 |
| **Ch12** | 0.282 | 0.427 | 0.681 | 0.384 | 0.657 | 0.256 | 0.524 | 0.344 | 0.573 | 0.414 | 0.816 |  | 0.788 | 0.437 | 0.395 | 0.401 | 0.553 | 0.434 | 0.585 |
| **Ch13** | 0.399 | 0.340 | 0.971 | 0.619 | 1.122 | 0.520 | 0.731 | 0.662 | 0.714 | 0.455 | 1.106 | 0.788 |  | 0.524 | 0.778 | 0.248 | 0.917 | 0.791 | 0.763 |
| **Ch14** | 0.353 | 0.226 | 0.592 | 0.756 | 0.923 | 0.626 | 0.619 | 0.721 | 0.500 | 0.485 | 0.494 | 0.437 | 0.524 |  | 1.031 | 0.788 | 0.611 | 0.583 | 0.439 |
| **Ch15** | 0.340 | 0.184 | 0.659 | 0.569 | 1.152 | 0.463 | 0.582 | 0.727 | 0.588 | 0.327 | 0.502 | 0.395 | 0.778 | 1.031 |  | 0.708 | 0.662 | 0.828 | 0.478 |
| **Ch16** | 0.171 | 0.136 | 0.354 | 0.312 | 0.568 | 0.253 | 0.287 | 0.551 | 0.368 | 0.317 | 0.249 | 0.401 | 0.248 | 0.788 | 0.708 |  | 0.326 | 0.611 | 0.280 |
| **Ch17** | 0.473 | 0.411 | 0.882 | 0.674 | 0.877 | 0.532 | 1.166 | 0.751 | 1.086 | 0.496 | 0.880 | 0.553 | 0.917 | 0.611 | 0.662 | 0.326 |  | 0.863 | 0.999 |
| **Ch18** | 0.253 | 0.184 | 0.679 | 0.536 | 0.849 | 0.495 | 0.650 | 1.214 | 0.568 | 0.361 | 0.691 | 0.434 | 0.791 | 0.583 | 0.828 | 0.611 | 0.863 |  | 0.576 |
| **Ch19** | 0.382 | 0.446 | 0.815 | 0.616 | 0.661 | 0.461 | 0.821 | 0.459 | 1.170 | 0.785 | 0.766 | 0.585 | 0.763 | 0.439 | 0.478 | 0.280 | 0.999 | 0.576 |  |

**Table S6 Data corresponding to brain network connections of Group 1 (Connectivity matrix: Fisher's z transformed values)**

Note: All values presented in this table are Fisher's z transformed values derived from original Pearson correlation coefficients. For intuitive interpretation of functional connectivity strength, back-transformed raw Pearson correlation coefficients are reported in Table 3. Group comparisons were performed using permutation tests, with all p-values corrected via the Benjamini–Hochberg false discovery rate (FDR) method.

**Table S7** **Data corresponding to brain network connections of Group 2 (Connectivity matrix: Fisher's z transformed values)**

|  | **Ch1** | **Ch2** | **Ch3** | **Ch4** | **Ch5** | **Ch6** | **Ch7** | **Ch8** | **Ch9** | **Ch10** | **Ch11** | **Ch12** | **Ch13** | **Ch14** | **Ch15** | **Ch16** | **Ch17** | **Ch18** | **Ch19** |
| --- | --- | --- | --- | --- | --- | --- | --- | --- | --- | --- | --- | --- | --- | --- | --- | --- | --- | --- | --- |
| **Ch1** |  | 0.858 | 0.308 | 0.234 | 0.170 | 0.415 | 0.275 | 0.300 | 0.224 | 0.345 | 0.281 | 0.343 | 0.162 | 0.283 | 0.177 | 0.074 | 0.277 | 0.191 | 0.275 |
| **Ch2** | 0.858 |  | 0.258 | 0.197 | 0.047 | 0.306 | 0.140 | 0.162 | 0.192 | 0.210 | 0.309 | 0.411 | 0.034 | 0.164 | 0.136 | 0.063 | 0.178 | 0.071 | 0.198 |
| **Ch3** | 0.308 | 0.258 |  | 0.812 | 0.663 | 0.525 | 0.832 | 0.527 | 0.560 | 0.358 | 0.766 | 0.271 | 0.702 | 0.317 | 0.235 | 0.113 | 0.552 | 0.443 | 0.552 |
| **Ch4** | 0.234 | 0.197 | 0.812 |  | 0.644 | 0.747 | 0.746 | 0.736 | 0.463 | 0.263 | 0.439 | 0.283 | 0.387 | 0.545 | 0.118 | 0.199 | 0.639 | 0.440 | 0.445 |
| **Ch5** | 0.170 | 0.047 | 0.663 | 0.644 |  | 0.552 | 0.702 | 0.663 | 0.411 | 0.241 | 0.400 | 0.280 | 0.792 | 0.664 | 0.714 | 0.487 | 0.562 | 0.596 | 0.421 |
| **Ch6** | 0.415 | 0.306 | 0.525 | 0.747 | 0.552 |  | 0.705 | 0.718 | 0.431 | 0.281 | 0.374 | 0.331 | 0.414 | 0.746 | 0.406 | 0.290 | 0.516 | 0.311 | 0.446 |
| **Ch7** | 0.275 | 0.140 | 0.832 | 0.746 | 0.702 | 0.705 |  | 0.702 | 0.695 | 0.382 | 0.515 | 0.332 | 0.559 | 0.460 | 0.310 | 0.271 | 0.892 | 0.461 | 0.593 |
| **Ch8** | 0.300 | 0.162 | 0.527 | 0.736 | 0.663 | 0.718 | 0.702 |  | 0.447 | 0.259 | 0.436 | 0.318 | 0.414 | 0.699 | 0.374 | 0.303 | 0.681 | 0.647 | 0.461 |
| **Ch9** | 0.224 | 0.192 | 0.560 | 0.463 | 0.411 | 0.431 | 0.695 | 0.447 |  | 0.796 | 0.462 | 0.261 | 0.393 | 0.282 | 0.075 | 0.117 | 0.966 | 0.323 | 0.913 |
| **Ch10** | 0.345 | 0.210 | 0.358 | 0.263 | 0.241 | 0.281 | 0.382 | 0.259 | 0.796 |  | 0.290 | 0.094 | 0.243 | 0.111 | 0.045 | 0.050 | 0.338 | 0.094 | 0.742 |
| **Ch11** | 0.281 | 0.309 | 0.766 | 0.439 | 0.400 | 0.374 | 0.515 | 0.436 | 0.462 | 0.290 |  | 0.671 | 0.752 | 0.311 | 0.179 | 0.143 | 0.455 | 0.374 | 0.423 |
| **Ch12** | 0.343 | 0.411 | 0.271 | 0.283 | 0.280 | 0.331 | 0.332 | 0.318 | 0.261 | 0.094 | 0.671 |  | 0.402 | 0.424 | 0.332 | 0.242 | 0.338 | 0.354 | 0.224 |
| **Ch13** | 0.162 | 0.034 | 0.702 | 0.387 | 0.792 | 0.414 | 0.559 | 0.414 | 0.393 | 0.243 | 0.752 | 0.402 |  | 0.371 | 0.524 | 0.128 | 0.537 | 0.456 | 0.458 |
| **Ch14** | 0.283 | 0.164 | 0.317 | 0.545 | 0.664 | 0.746 | 0.460 | 0.699 | 0.282 | 0.111 | 0.311 | 0.424 | 0.371 |  | 0.849 | 0.832 | 0.442 | 0.607 | 0.349 |
| **Ch15** | 0.177 | 0.136 | 0.235 | 0.118 | 0.714 | 0.406 | 0.310 | 0.374 | 0.075 | 0.045 | 0.179 | 0.332 | 0.524 | 0.849 |  | 0.728 | 0.239 | 0.545 | 0.251 |
| **Ch16** | 0.074 | 0.063 | 0.113 | 0.199 | 0.487 | 0.290 | 0.271 | 0.303 | 0.117 | 0.050 | 0.143 | 0.242 | 0.128 | 0.832 | 0.728 |  | 0.156 | 0.619 | 0.261 |
| **Ch17** | 0.277 | 0.178 | 0.552 | 0.639 | 0.562 | 0.516 | 0.892 | 0.681 | 0.966 | 0.338 | 0.455 | 0.338 | 0.537 | 0.442 | 0.239 | 0.156 |  | 0.611 | 0.766 |
| **Ch18** | 0.191 | 0.071 | 0.443 | 0.440 | 0.596 | 0.311 | 0.461 | 0.647 | 0.323 | 0.094 | 0.374 | 0.354 | 0.456 | 0.607 | 0.545 | 0.619 | 0.611 |  | 0.478 |
| **Ch19** | 0.275 | 0.198 | 0.552 | 0.445 | 0.421 | 0.446 | 0.593 | 0.461 | 0.913 | 0.742 | 0.423 | 0.224 | 0.458 | 0.349 | 0.251 | 0.261 | 0.766 | 0.478 |  |

Note: All values presented in this table are Fisher's z transformed values derived from original Pearson correlation coefficients. For intuitive interpretation of functional connectivity strength, back-transformed raw Pearson correlation coefficients are reported in Table 3. Group comparisons were performed using permutation tests, with all p-values corrected via the Benjamini–Hochberg false discovery rate (FDR) method.

**Table S8 Shapiro–Wilk normality test results for all continuous variables**

| Variable | Shapiro–Wilk Statistic | df | *p* | Distribution |
| --- | --- | --- | --- | --- |
| Age | 0.885 | 64 | <0.001 | Non-normal |
| FSIQ | 0.898 | 64 | <0.001 | Non-normal |
| Correct words per minute | 0.898 | 64 | <0.001 | Non-normal |
| CARS | 0.945 | 64 | 0.006 | Non-normal |
| Oxy-Hb Ch1 | 0.973 | 64 | 0.164 | Normal |
| Oxy-Hb Ch2 | 0.990 | 64 | 0.901 | Normal |
| Oxy-Hb Ch3 | 0.763 | 64 | <0.001 | Non-normal |
| Oxy-Hb Ch4 | 0.859 | 64 | <0.001 | Non-normal |
| Oxy-Hb Ch5 | 0.912 | 64 | <0.001 | Non-normal |
| Oxy-Hb Ch6 | 0.813 | 64 | <0.001 | Non-normal |
| Oxy-Hb Ch7 | 0.882 | 64 | <0.001 | Non-normal |
| Oxy-Hb Ch8 | 0.929 | 64 | 0.001 | Non-normal |
| Oxy-Hb Ch9 | 0.570 | 64 | <0.001 | Non-normal |
| Oxy-Hb Ch10 | 0.684 | 64 | <0.001 | Non-normal |
| Oxy-Hb Ch11 | 0.951 | 64 | 0.012 | Non-normal |
| Oxy-Hb Ch12 | 0.777 | 64 | <0.001 | Non-normal |
| Oxy-Hb Ch13 | 0.946 | 64 | 0.007 | Non-normal |
| Oxy-Hb Ch14 | 0.899 | 64 | <0.001 | Non-normal |
| Oxy-Hb Ch15 | 0.797 | 64 | <0.001 | Non-normal |
| Oxy-Hb Ch16 | 0.912 | 64 | <0.001 | Non-normal |
| Oxy-Hb Ch17 | 0.974 | 64 | 0.190 | Normal |
| Oxy-Hb Ch18 | 0.928 | 64 | 0.001 | Non-normal |
| Oxy-Hb Ch19 | 0.933 | 64 | 0.002 | Non-normal |
| HBR Ch1 | 0.843 | 64 | <0.001 | Non-normal |
| HBR Ch2 | 0.917 | 64 | <0.001 | Non-normal |
| HBR Ch3 | 0.348 | 64 | <0.001 | Non-normal |
| HBR Ch4 | 0.408 | 64 | <0.001 | Non-normal |
| HBR Ch5 | 0.702 | 64 | <0.001 | Non-normal |
| HBR Ch6 | 0.660 | 64 | <0.001 | Non-normal |
| HBR Ch7 | 0.803 | 64 | <0.001 | Non-normal |
| HBR Ch8 | 0.685 | 64 | <0.001 | Non-normal |
| HBR Ch9 | 0.618 | 64 | <0.001 | Non-normal |
| HBR Ch10 | 0.757 | 64 | <0.001 | Non-normal |
| HBR Ch11 | 0.777 | 64 | <0.001 | Non-normal |
| HBR Ch12 | 0.628 | 64 | <0.001 | Non-normal |
| HBR Ch13 | 0.766 | 64 | <0.001 | Non-normal |
| HBR Ch14 | 0.819 | 64 | <0.001 | Non-normal |
| HBR Ch15 | 0.783 | 64 | <0.001 | Non-normal |
| HBR Ch16 | 0.750 | 64 | <0.001 | Non-normal |
| HBR Ch17 | 0.891 | 64 | <0.001 | Non-normal |
| HBR Ch18 | 0.688 | 64 | <0.001 | Non-normal |
| HBR Ch19 | 0.840 | 64 | <0.001 | Non-normal |

*Note*. df = degrees of freedom. Normal distribution was defined as *p* > 0.05.

**Table S9 Spearman correlations of oxygenated and deoxygenated hemoglobin signals with demographic and clinical variables**

| Variable | Age (rₛ) | *p* | FSIQ (rₛ) | *p* | Gender (rₛ) | *p* | Only child (rₛ) | *p* | Parental care (rₛ) | *p* | Correct words (rₛ) | *p* | CARS (rₛ) | *p* |
| --- | --- | --- | --- | --- | --- | --- | --- | --- | --- | --- | --- | --- | --- | --- |
| Oxy-Hb Ch1 | 0.084 | 0.507 | 0.304 | **0.015** | −0.024 | 0.849 | 0.062 | 0.629 | 0.116 | 0.362 | 0.529 | **<0.001** | 0.003 | 0.979 |
| Oxy-Hb Ch2 | 0.178 | 0.160 | 0.405 | **0.001** | 0.136 | 0.284 | 0.221 | 0.079 | 0.095 | 0.456 | 0.569 | **<0.001** | −0.045 | 0.726 |
| Oxy-Hb Ch3 | 0.170 | 0.178 | −0.059 | 0.644 | −0.010 | 0.939 | 0.247 | **0.049** | 0.076 | 0.552 | −0.002 | 0.988 | 0.089 | 0.485 |
| Oxy-Hb Ch4 | 0.178 | 0.158 | 0.042 | 0.743 | −0.005 | 0.970 | 0.330 | **0.008** | 0.171 | 0.176 | 0.005 | 0.968 | 0.083 | 0.517 |
| Oxy-Hb Ch5 | 0.199 | 0.116 | 0.062 | 0.628 | −0.005 | 0.970 | 0.356 | **0.004** | 0.081 | 0.523 | 0.074 | 0.560 | −0.089 | 0.484 |
| Oxy-Hb Ch6 | 0.157 | 0.214 | −0.003 | 0.984 | −0.122 | 0.339 | 0.192 | 0.129 | 0.250 | **0.046** | −0.053 | 0.679 | 0.105 | 0.407 |
| Oxy-Hb Ch7 | 0.122 | 0.337 | 0.114 | 0.371 | 0.039 | 0.760 | 0.365 | **0.003** | 0.043 | 0.735 | 0.083 | 0.514 | 0.109 | 0.390 |
| Oxy-Hb Ch8 | 0.178 | 0.160 | 0.066 | 0.604 | 0.024 | 0.849 | 0.263 | **0.036** | −0.030 | 0.816 | −0.031 | 0.810 | −0.047 | 0.712 |
| Oxy-Hb Ch9 | 0.212 | 0.093 | −0.004 | 0.974 | −0.005 | 0.970 | 0.178 | 0.160 | 0.083 | 0.513 | 0.038 | 0.766 | 0.046 | 0.717 |
| Oxy-Hb Ch10 | 0.174 | 0.170 | 0.253 | **0.043** | 0.107 | 0.400 | 0.169 | 0.182 | 0.043 | 0.735 | 0.290 | **0.020** | 0.067 | 0.601 |
| Oxy-Hb Ch11 | 0.160 | 0.207 | 0.114 | 0.372 | 0.287 | **0.022** | 0.167 | 0.186 | −0.095 | 0.456 | 0.234 | 0.063 | −0.024 | 0.853 |
| Oxy-Hb Ch12 | 0.013 | 0.921 | 0.053 | 0.679 | 0.053 | 0.675 | 0.207 | 0.100 | 0.131 | 0.301 | 0.198 | 0.116 | −0.195 | 0.122 |
| Oxy-Hb Ch13 | 0.323 | **0.009** | 0.088 | 0.487 | 0.243 | 0.053 | 0.303 | **0.015** | −0.030 | 0.816 | 0.116 | 0.361 | 0.025 | 0.843 |
| Oxy-Hb Ch14 | 0.183 | 0.148 | 0.164 | 0.195 | 0.039 | 0.760 | 0.287 | **0.022** | 0.076 | 0.552 | 0.046 | 0.716 | −0.135 | 0.289 |
| Oxy-Hb Ch15 | 0.193 | 0.127 | 0.059 | 0.642 | −0.107 | 0.400 | 0.264 | **0.035** | 0.087 | 0.494 | −0.005 | 0.968 | 0.086 | 0.500 |
| Oxy-Hb Ch16 | 0.028 | 0.829 | −0.069 | 0.589 | −0.029 | 0.819 | 0.001 | 0.995 | 0.189 | 0.135 | 0.024 | 0.852 | −0.053 | 0.675 |
| Oxy-Hb Ch17 | 0.118 | 0.355 | 0.001 | 0.997 | 0.053 | 0.675 | 0.306 | **0.014** | 0.091 | 0.475 | 0.006 | 0.962 | 0.071 | 0.577 |
| Oxy-Hb Ch18 | 0.042 | 0.742 | −0.000 | 0.999 | 0.044 | 0.731 | 0.214 | 0.089 | 0.079 | 0.532 | −0.059 | 0.646 | 0.065 | 0.607 |
| Oxy-Hb Ch19 | 0.071 | 0.579 | 0.090 | 0.479 | −0.058 | 0.647 | 0.212 | 0.092 | 0.141 | 0.267 | 0.014 | 0.914 | 0.227 | 0.071 |
| HBR Ch1 | 0.010 | 0.935 | −0.146 | 0.250 | −0.219 | 0.082 | 0.153 | 0.226 | 0.127 | 0.316 | −0.161 | 0.205 | −0.003 | 0.981 |
| HBR Ch2 | 0.186 | 0.140 | −0.159 | 0.211 | −0.005 | 0.970 | 0.205 | 0.103 | 0.112 | 0.378 | −0.075 | 0.554 | 0.013 | 0.919 |
| HBR Ch3 | 0.014 | 0.912 | −0.258 | **0.040** | −0.170 | 0.179 | −0.240 | 0.056 | 0.045 | 0.724 | −0.130 | 0.307 | −0.055 | 0.664 |
| HBR Ch4 | −0.090 | 0.479 | −0.165 | 0.193 | −0.015 | 0.909 | −0.103 | 0.417 | 0.112 | 0.378 | −0.097 | 0.445 | 0.034 | 0.792 |
| HBR Ch5 | −0.043 | 0.739 | 0.034 | 0.788 | −0.122 | 0.339 | −0.145 | 0.254 | −0.116 | 0.362 | 0.164 | 0.195 | −0.061 | 0.630 |
| HBR Ch6 | −0.126 | 0.323 | −0.158 | 0.214 | −0.180 | 0.155 | −0.124 | 0.329 | 0.198 | 0.116 | −0.187 | 0.138 | −0.008 | 0.950 |
| HBR Ch7 | −0.098 | 0.442 | −0.358 | **0.004** | −0.092 | 0.468 | −0.065 | 0.610 | 0.187 | 0.140 | −0.049 | 0.702 | 0.000 | 1.000 |
| HBR Ch8 | −0.123 | 0.332 | −0.140 | 0.270 | 0.088 | 0.492 | −0.339 | **0.006** | −0.039 | 0.758 | 0.050 | 0.693 | −0.134 | 0.289 |
| HBR Ch9 | −0.127 | 0.317 | −0.220 | 0.080 | −0.141 | 0.267 | −0.122 | 0.336 | 0.020 | 0.875 | −0.084 | 0.510 | −0.035 | 0.785 |
| HBR Ch10 | 0.049 | 0.701 | −0.247 | **0.049** | −0.010 | 0.939 | 0.029 | 0.822 | −0.129 | 0.309 | −0.160 | 0.207 | 0.065 | 0.609 |
| HBR Ch11 | 0.038 | 0.766 | −0.037 | 0.773 | 0.190 | 0.133 | −0.119 | 0.350 | −0.215 | 0.087 | 0.154 | 0.223 | −0.163 | 0.197 |
| HBR Ch12 | 0.046 | 0.718 | 0.049 | 0.701 | 0.044 | 0.731 | 0.060 | 0.639 | −0.154 | 0.224 | 0.178 | 0.160 | −0.227 | 0.072 |
| HBR Ch13 | −0.000 | 0.998 | 0.085 | 0.505 | 0.126 | 0.320 | −0.034 | 0.791 | −0.231 | 0.067 | 0.250 | **0.046** | −0.090 | 0.480 |
| HBR Ch14 | −0.035 | 0.785 | −0.029 | 0.818 | 0.194 | 0.124 | 0.155 | 0.221 | 0.012 | 0.922 | −0.063 | 0.620 | −0.056 | 0.659 |
| HBR Ch15 | 0.123 | 0.335 | 0.024 | 0.853 | 0.122 | 0.339 | 0.065 | 0.610 | −0.114 | 0.370 | 0.048 | 0.704 | 0.028 | 0.824 |
| HBR Ch16 | 0.021 | 0.871 | −0.035 | 0.782 | 0.015 | 0.909 | −0.023 | 0.854 | 0.037 | 0.770 | −0.067 | 0.597 | −0.006 | 0.961 |
| HBR Ch17 | −0.270 | **0.031** | −0.117 | 0.355 | −0.005 | 0.970 | −0.134 | 0.290 | 0.116 | 0.362 | 0.157 | 0.217 | 0.040 | 0.755 |
| HBR Ch18 | −0.175 | 0.168 | −0.156 | 0.219 | −0.019 | 0.879 | −0.282 | **0.024** | −0.016 | 0.898 | 0.021 | 0.872 | −0.123 | 0.334 |
| HBR Ch19 | −0.122 | 0.336 | −0.217 | 0.085 | −0.185 | 0.144 | −0.126 | 0.322 | 0.039 | 0.758 | −0.057 | 0.652 | 0.257 | 0.041 |

*Note.* All analyses used Spearman’s rank correlation (*r*ₛ) because most variables were non-normally distributed. Oxy-Hb = oxygenated hemoglobin; HBR = deoxygenated hemoglobin. *p* < 0.05 was considered statistically significant.
